# Characterization of a submicro-X-ray fluorescence setup on the B16 beamline at Diamond Light Source

**DOI:** 10.1107/S1600577518006203

**Published:** 2018-06-13

**Authors:** M. Rauwolf, A. Turyanskaya, D. Ingerle, N. Szoboszlai, I. Pape, A. W. Malandain, O. J. L. Fox, L. Hahn, K. J. S. Sawhney, C. Streli

**Affiliations:** a Atominstitut, TU Wien, Vienna, Austria; b Laboratory of Environmental Chemistry and Bioanalytics, Department of Analytical Chemistry, Institute of Chemistry, Eötvös Loránd University, Budapest, Hungary; c Diamond Light Source Ltd, Harwell Science and Innovation Campus, Didcot, UK; d Karlsruhe Institute of Technology (KIT), Karlsruhe, Germany

**Keywords:** SR submicro-XRF, beamline B16, elemental imaging, scanning, human bone, cells

## Abstract

An X-ray fluorescence setup has been tested on the B16 beamline at the Diamond Light Source synchrotron with two different excitation energies (12.7 and 17 keV). This setup allows the scanning of thin samples (thicknesses up to several micrometers) with a sub-micrometer resolution (beam size of 500 nm × 600 nm determined with a 50 µm Au wire). Sensitivities and detection limits reaching values of 249 counts s^−1^ fg^−1^ and 4 ag in 1000 s, respectively (for As *K*α excited with 17 keV), are presented in order to demonstrate the capabilities of this setup. Sample measurements of a human bone and a single cell performed at B16 are presented in order to illustrate the suitability of the setup in biological applications.

## Abstract 2

The use of the X-ray fluorescence setup on the B16 beamline at the Diamond Light Source for X-ray imaging with sub-micrometer resolution was described and it was shown that this setup is very well suited for studying thin samples, especially for biological applications.

## 1. Introduction   

Synchrotron-radiation-induced X-ray fluorescence (SR-XRF) analysis with micro- or nanometer resolution is a powerful method for non-destructively investigating element distribution in a wide variety of samples (Janssens *et al.*, 2010 ▸; Holt *et al.*, 2013 ▸; West *et al.*, 2017 ▸). SR-XRF imaging can be performed in scanning or in full-field mode. While the ideal analysis mode depends on the elements of interest and their concentration, in our experience imaging of trace elements works best in scanning mode. In our previous study we demonstrated that, in bone, Ca (the major element of bone) was nicely imaged with both a full-field color X-ray camera setup at the BAMline at BESSY II and at the scanning confocal micro-XRF setup at the FLUO beamline at ANKA. However, whereas Zn (a trace element in bone) was quite easy to image in scanning mode, it was barely possible to detect in full-field mode. Therefore, the scanning mode is our preferred choice for imaging bone samples (Rauwolf *et al.*, 2017 ▸). Nowadays, the smallest beam sizes (and therefore highest resolution) available for scanning SR-XRF imaging reach a few tens of nanometers (Martínez-Criado *et al.*, 2016 ▸; Lemelle *et al.*, 2017 ▸). However, for many biological samples such high-resolution beams are already too small to scan meaningful areas of interest on the samples on a reasonable time scale. For instance, the samples of our interest are bone tissue and cells. Within the bone we are mostly interested in osteons (diameter of 200 µm). Diameters of the cells can vary between 10 and 120 µm. As one can imagine, scanning such an object with such a small beam would require considerable time. Therefore, submicro-beam would be a perfect choice, allowing measurements of such structures and, moreover, allowing resolving of substructures, such as cement lines in bone (several micrometers thick). To find the ideal SR-XRF imaging method for the investigation of trace element distributions and possible substructures (in the micrometer range) in biological samples, such as bone, single cells and other tissues (Ugarte *et al.*, 2016 ▸; Mihucz *et al.*, 2016 ▸), SR-XRF at the B16 beamline at Diamond Light Source was tested with different samples and standards. Test measurements were performed with two different excitation energies, 12.7 and 17 keV.

## 2. Materials and methods   

### 2.1. SR-XRF setup   

**Figure 1:**
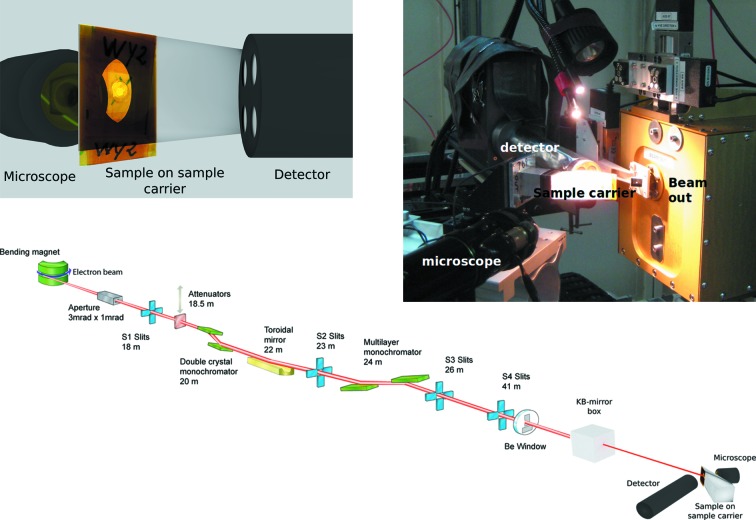
Top left: image of the microscope, a sample on its carrier and the detector (seen from the position of the beam). Top right: photograph of the setup at B16. Bottom: B16 beamline schematic. [Image adapted from Diamond (2017*b* ▸).]

The SR-XRF setup was installed for multiple experimental sessions at the B16 bending magnet test beamline of the Diamond Light Source synchrotron (see Fig. 1 ▸), a beamline committed to be easily adaptable and adjustable to the particular requirements of various experiments (Sawhney *et al.*, 2010 ▸, 2011 ▸). The Diamond Light Source storage ring operates in top-up mode (3 GeV electron energy, 300 mA ring current). For the SR submicro-XRF experiment, the X-ray energy was set with an RuB_4_C double-multilayer monochromator (FMB Oxford Ltd, Oxford, UK), which can be used to produce a high-flux monochromatic beam in the range 8–20 keV (Diamond, 2017*a* ▸). The beam was focused with a Kirkpatrick–Baez (KB) mirror pair (Diamond Light Source Ltd, in-house design and construction). The sample stage consisted of piezo crystal-based motors (each of the *xyz* axes is an ECS5050 piezo stage; Attocube Systems AG, Munich, Germany) and a high-purity methyl acrylate sample holder, which was 3D-printed from a clear photoreactive resin (Form 2, Formlabs Inc., Somerville, USA) at Diamond Light Source. The angle between the primary beam and sample was 45°. Additionally, the setup was equipped with an optical microscope (Optem Fusion, Qioptiq), which was focused onto the back side of the samples (see Fig. 1 ▸). This allowed identification of the regions of interest quickly. The XRF radiation was detected at an angle of 45° to the sample or 90° to the primary beam. Note that, due to the setup’s geometry, in particular the angle of 45° between primary beam and sample, the horizontal resolution worsens with increasing sample thickness. Additionally, as the microscope sees the sample from the back side, the sample should be transparent in order to find the areas of interest. Therefore, the recommended sample thickness should be in a range up to several micrometers.

The size of the focused beam was determined using a knife-edge scan of a 50 µm Au wire. Measurements of standards were performed at 17 keV and 12.7 keV to optimize the excitation for different elements (once for elements up to Sr and once for those up to Zn).

#### 2.1.1. Setup for 17 keV   

The 17 keV measurements were recorded with a Vortex four-element detector (Vortex-ME4, Hitachi Ltd, Tokyo, Japan) with a total active area of 200 µm^2^ and SII electronics. The beam resolution (full width at half-maximum, FWHM) determined with a 50 µm Au cross was about 500 nm (vertical) × 600 nm (horizontal).

**Figure 2:**
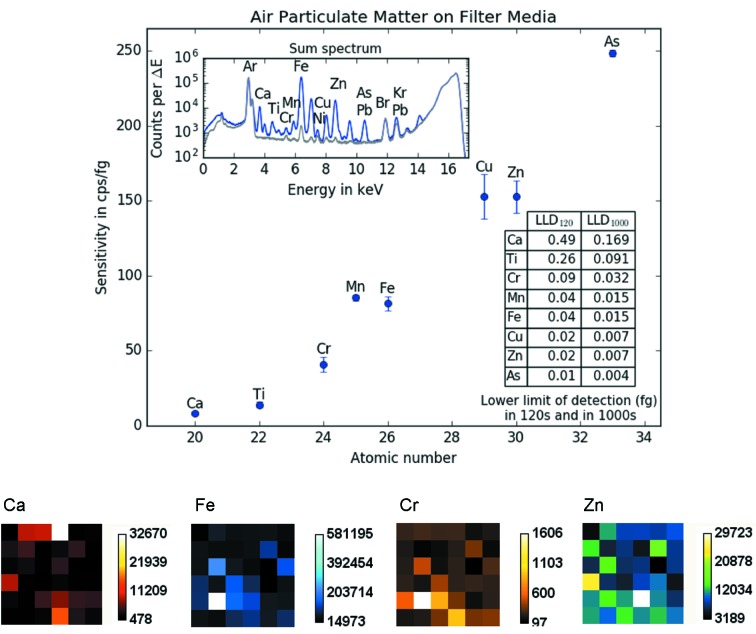
Top: sensitivities, lower limits of detection (inset table) and sum spectra (inset; in blue for the loaded, gray for the blank filter) of 36 (6 × 6) pixels measured on the NIST Standard Reference Material (SRM) 2783 (Air Particulate on Filter Media) with 17 keV monochromatic excitation. Note that the error bars on the sensitivity values are most likely underestimated due to the small size of the measured area. Bottom: distribution maps for four selected elements (Ca, Fe, Cr and Zn) showing the inhomogeneity of the SRM. The distance between pixels was 10 µm. The measurement time per pixel was 120 s.

To characterize the setup, especially for thin samples, a NIST standard (Standard Reference Material 2783 – Air Particulate on Filter Media; NIST, Gaithersburg, USA) was measured. This standard is quite heterogeneous as one can see from the distribution maps of Ca, Fe, Cr and Zn in Fig. 2 ▸. The certified values can only be considered to be valid within the stated uncertainty if an area of at least 1 cm^2^ is measured (NIST, 2011 ▸). As one can easily see, this is too large an area to analyze with a sub-micrometer beam. Additionally, a measurement time of 120 s per pixel was required for this standard to produce an evaluable spectrum. To obtain sensitivity and lower limits of detection in reasonable time, the area scanned was limited to 6 × 6 pixels (considering our beam size this results in an area of about 10.8 µm^2^). Between each point measured the sample was moved by 10 µm (step size). Additionally to the loaded filter, a blank filter (also part of the NIST SRM 2783) was scanned in the same way. This allowed determination of the components of the signal that originate from the blank filter alone. Sum spectra for the loaded and the blank filters (see Fig. 2 ▸, top left) were fitted using *AXIL-QXAS*, version 3.6 (IAEA, 2009 ▸). Sensitivities and lower limits of detection (LLD) were calculated using the fitted result of the loaded filter corrected with the net counts of the blank filter. The uncertainties for the sensitivities were calculated by using the uncertainties (about 95%) given in the NIST certificate (NIST, 2011 ▸) as well as two times the fit error (2σ) of the fitted net counts of the loaded filter. Note that, due to the considerably smaller measured area (compared with the given recommendation), the error bars shown in Fig. 2 ▸ do not correspond to a confidence interval of 95% and are only given as a rough indication. As one can see from Fig. 2 ▸, the sensitivities obtained ranged from about 8 counts s^−1^ fg^−1^ for Ca *K*α to 249 counts s^−1^ fg^−1^ for As *K*α, while lower limits of detection ranged from 169 to 4 ag in 1000 s. Sensitivity values shown in Fig. 2 ▸ can also be found in Table S1 of the supporting information.

To put these values into perspective, a comparison with the LLDs for Mn and Cu reached at the ID16B-NA beamline at the ESRF is given in Table 1 ▸.

#### 2.1.2. Setup for 12.7 keV   

The measurements with an excitation energy of 12.7 keV were performed with a different detector, a single silicon drift detector (SDD) (Vortex 90EX, Hitachi Ltd, Tokyo, Japan; 50 mm^2^ active area; Canberra counting electronics). The beam resolution (FWHM) was also about 500 nm (vertical) × 600 nm (horizontal).

**Figure 3:**
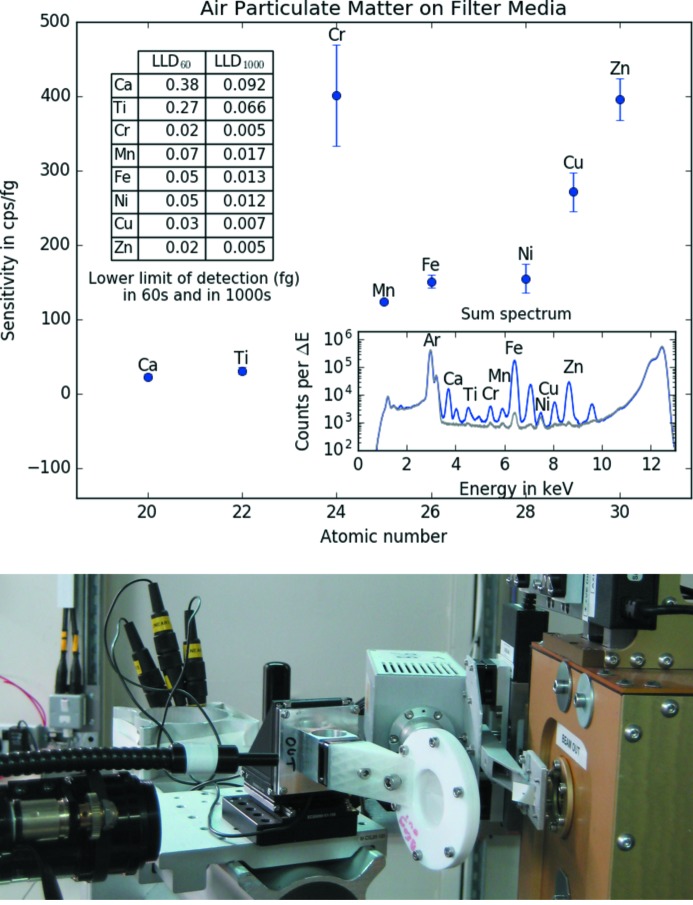
Top: sensitivities, lower limits of detection (inset table) and sum spectra (inset; in blue for the loaded, gray for the blank filter) of 41 pixels measured on the NIST Standard Reference Material 2783 (Air Particulate on Filter Media) with 12.7 keV monochromatic excitation. Bottom: photograph of the NIST SRM 2783 in the XRF setup on B16.

With the same step size and measurement time as described in §2.1.1, the NIST standard SRM 2783 (both loaded and blank filters) (NIST, 2011 ▸) was also measured with 12.7 keV excitation energy. In total, 41 spectra (41 × 1 pixels) were recorded and the results are shown in Fig. 3 ▸. The sensitivities obtained ranged from about 23 counts s^−1^ fg^−1^ for Ca *K*α to 396 counts s^−1^ fg^−1^ for Zn *K*α while lower limits of detection ranged from 92 to 5 ag in 1000 s. For the 12.7 keV excitation it was also possible to obtain sensitivity and LLD values for Ni *K*α. This was not possible for the 17 keV excitation due to the contributions to the Ni signal from the blank filter and the lower sensitivities. The high sensitivity and LLD values for Cr *K*α are caused by a single Cr hotspot in the scan. In this hotspot Cr reaches 16408 counts in 60 s while all other pixels in the scan have values between 22 and 1046 counts in 60 s. The sensitivity values presented in Fig. 3 ▸ can also be found in Table S2 of the supporting information.

**Figure 4:**
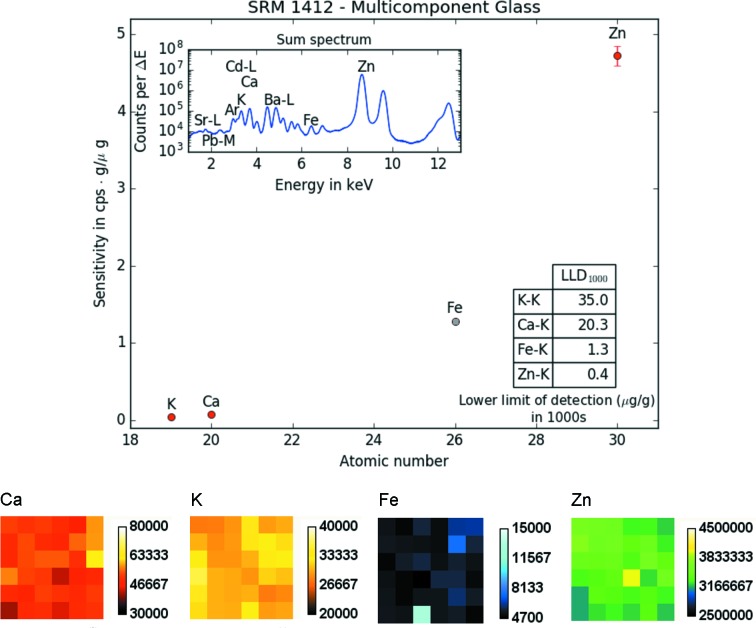
Top: sensitivities for the *K*-lines, lower limits of detection (inset table) and sum spectrum (inset) of 36 (6 × 6) pixels measured on the NIST Standard Reference Material 1412 (Multicomponent Glass) with 12.7 keV monochromatic excitation. Iron is marked in gray as it is just a reference and not a certified value. Bottom: distribution maps for Ca, K, Fe and Zn showing the homogeneity of the SRM. The distance between pixels was 10 µm. The measurement time per pixel was 20 s.

To further characterize the B16 submicro-XRF setup, sensitivities and LLD (in µg g^−1^) were also determined for a thick homogeneous standard reference material (NIST SRM 1412 – Multicomponent Glass; NIST, Gaithersburg, USA) (National Bureau of Standards, 1985 ▸). Again, a scan area of 6 × 6 pixels was measured (with a step size of 10 µm). Measurement time was 20 s. Sensitivities for *K*- and *L*-lines [in counts s^−1^ (µg g^−1^)^−1^] as well as the sum spectrum and the LLD (µg g^−1^ in 1000 s) are shown in Figs. 4 ▸ and 5 ▸, respectively.

A comparison of the LLDs for Ca, Fe and Cu reached at the P06 beamline at PETRA III can be seen in Table 2 ▸.

## 3. Scanned samples – results   

To show the potential of the XRF setup on B16, scans from three projects are presented in this section. The analysis of three different sample types was carried out at 17 keV. An Au test structure was measured to show the resolution capabilities of the setup. The results of an area measured in bone and the elemental maps gained by measuring a single cell demonstrate the potential of the XRF setup tested at B16 for biological samples.

### 3.1. Gold test structure   

**Figure 6:**
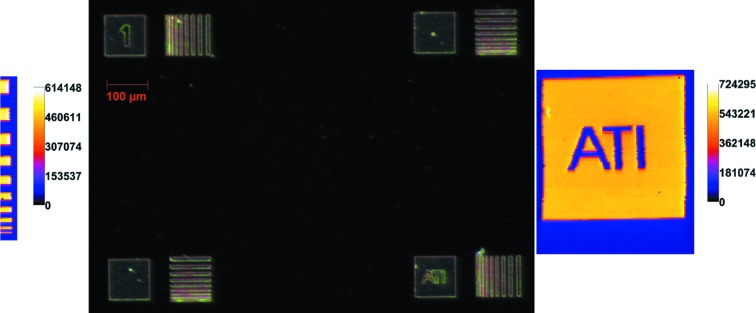
Au maps from SR-XRF (left and right) and microscope image (middle) of the KNMF gold test structure.

This sample was produced for the Atominstitut (ATI) by the Karlsruhe Nano Micro Facility (KNMF) by means of electron beam lithography. A four-inch silicon wafer was coated with a start layer (10 nm Ti, 100 nm Au) for electroplating by vapor deposition. The structures were written as trenches in a 1.5 µm-thick PMMA layer with an electron beam writer (EBPG5200Z, Raith). The dose was 800 µC cm^−2^. Development took place in a mixture of MIBK and IPA (1:1). The trenches had been filled with gold of thickness 1 mm by electroplating. Residual resist was removed by oxygen plasma. The sample is a Ti-coated (over the whole surface) Si wafer. On top of the Ti layer there are Au structures in the shape of 100 µm × 100 µm squares and consecutive stripes of different widths between 1 and 10 µm. A microscope image of the sample is shown in Fig. 6 ▸.

Two areas of this sample were scanned with the XRF setup on B16: the square shown at the bottom right (of the microscope image, Fig. 6 ▸) as well as parts of the line structure on the bottom left. From top to bottom, the first stripe of the line structure has a width of 10 µm and is followed by a gap of 10 µm; the second stripe has a width of 9 µm, followed by a 9 µm gap. The stripe widths and stripe gaps decrease in 1 µm steps down to the smallest stripe which has a width of 1 µm. Both structures were scanned with a step size of 1 µm and a measurement time of 1 s. Measurements for the two Au maps took about 50 min for the stripes and 8 h for the square. The time given above includes the time needed for the communication of the measurement software with the detector and motor movements time (about 1 s per pixel). The results from the scanned Au structure confirm that 1 µm structures are easily resolved with the XRF setup on B16.

### 3.2. Human bone biopsy sample   

The sample is a vertebral body biopsy and was prepared as a 4 µm (thickness) cut. This cut was sandwiched between two 8 µm Kapton foils and fastened between Plexiglas frames. The bone sample was thus fixed on the sample carrier, which can be seen in Fig. 1 ▸.

**Figure 7:**
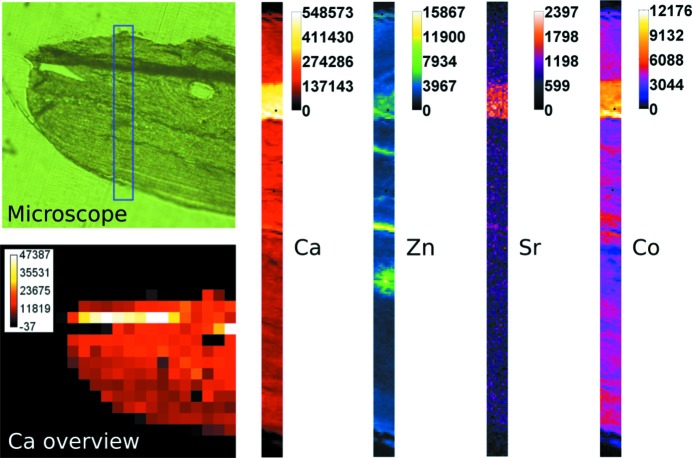
Microscope image (upper left) of the measured bone sample as well as the Ca distribution of a rough scan over this sample (lower left) and the distributions for Ca, Zn, Sr and Co of the fine scan (right).

Fig. 7 ▸ shows a microscope image of the area of the sample measured (top left), the Ca distribution of an overview scan (bottom left) and the Ca, Zn, Sr and Co distribution of the final high-resolution scan. The overview scan was performed with a step size of 20 µm and a measurement time per pixel of 2 s. The overview area was 400 µm × 360 µm and the scan took about 20 min.

The high-resolution scan (step size 1 µm) was performed in the area marked with a blue frame in the microscope image and was optimized for measuring Zn and therefore a considerably longer measurement time (20 s per pixel) was needed. The whole scan took about 15 h. The scan area was 11 µm × 237 µm. Fig. 7 ▸ demonstrates that the setup is not only capable of imaging thin structures in bone samples (especially visible in the Zn map) but also that the resulting elemental maps can easily be correlated to microscope images using rough scans.

### 3.3. Cancer cell   

These measurements were performed as part of a project investigating the localization of metals within cells. Human A-375 malignant melanoma cells (ECACC, Salisbury, UK) were grown on 7.5 mm × 7.5 mm low-stress silicon nitride windows with a thickness of 500 nm (Norcada, Edmonton, AB, Canada). Cells were treated with copper sulfate and chelators. A more detailed description of the sample treatment is given by Gaál *et al.* (2018*a* ▸,*b* ▸).

Generally, a maximum of 100 cells per total area is sufficient. Usually a cell suspension of half a million cells in 2 ml of medium is prepared and applied to the plate. The cells will adhere to the membrane. If too many cells have been placed on the membrane, the process should be started from the beginning; if too little, then more cells should be pipetted. If this method is used, we can scan individual cells and small groups of cells on the same membrane.

**Figure 8:**
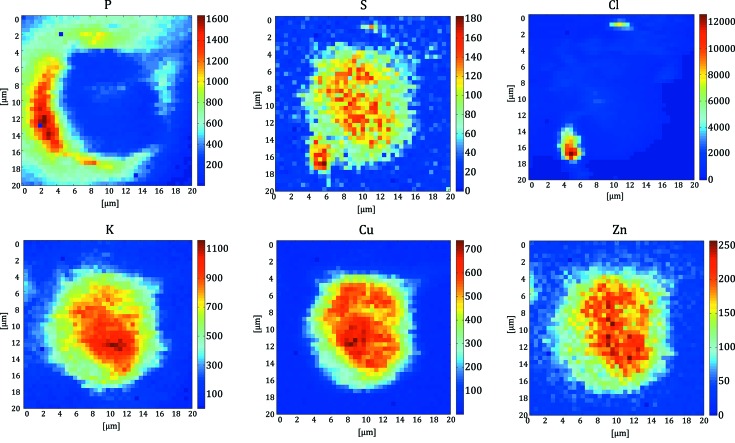
P, S, Cl, K, Cu and Zn maps of a copper-poisoned human A-375 malignant melanoma cell.

The cell sample area was measured for 5 s per pixel with a step size of 0.5 µm. The area size was 20 µm × 20 µm (41 × 41 pixels). The complete scan took about 2.8 h. The elemental maps in Fig. 8 ▸ show the suitability of the setup for investigating elemental distributions within a single cell.

## 4. Conclusion   

We characterized an XRF setup on beamline B16 at Diamond Light Source using two different excitation energies (12.7 and 17 keV) as well as two different standard samples. The beam size was determined as 500 nm × 600 nm and thus the 1 µm structures of the Au test sample were easily resolved. For the excitation energy of 17 keV the following sensitivities were obtained: 8 counts s^−1^ fg^−1^ for Ca *K* to 249 counts s^−1^ fg^−1^ for As *K*. The lower limits of detection ranged from 169 ag (Ca) to 4 ag (As) in 1000 s. For 12 keV, sensitivities obtained ranged from about 23 counts s^−1^ fg^−1^ for Ca *K* to 396 counts s^−1^ fg^−1^ for Zn *K*. For the lower limits of detection, values from 92 ag (Ca) to 5 ag (Zn) in 1000 s were achieved. Although the detection limits presented do not reach the impressive values of the P06 beamline at PETRA III (due to the considerably lower flux) and of ID16B-NA at ESRF (due to the larger beam size), the setup is very recommendable for imaging biological samples, which we have illustrated by means of scans on a bone sample and a single cancer cell. Additionally, it should be mentioned that at Diamond Light Source there exists the possibility to first scan a larger sample area at I18 with micrometer resolution (for example 2 × 2 µm) followed by further investigation of smaller areas of interest with sub-micrometer resolution at B16 (Ugarte *et al.*, 2016 ▸). In conclusion, the XRF setup on the B16 beamline is very well suited for studying thin samples and especially for biological applications.

## Supplementary Material

Tables S1 and S2: Calculation of the sensitivities for 17 keV and 12.7 keV. DOI: 10.1107/S1600577518006203/pp5117sup1.pdf

## Figures and Tables

**Figure 5:**
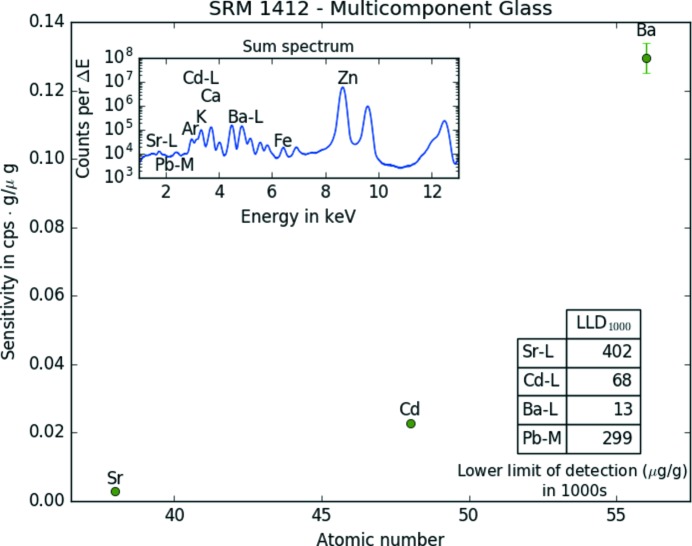
Sensitivities for the *L*-lines, lower limit of detections (inset table) and sum spectrum (inset) of 36 (6 × 6) pixels measured on the NIST SRM 1412 (Multicomponent Glass) with 12.7 keV monochromatic excitation.

**Table 1:** Comparision of the lower limits of detection (LLD) for Mn and Cu determined for the B16 beamline at Diamond Light Source and the ID16B-NA beamline at the ESRF as well as some of the measurement parameters For both beamlines a multi-element standard was scanned to determine the LLDs for various elements. The values for the ESRF ID16-NA beamline are taken from Laforce *et al.* (2014 ▸).

	Diamond B16	ESRF ID16B-NA
Mn *K* LLD (in ag for 1000 s)	15	0.0457 (12 p.p.m.)
Cu *K* LLD (in ag for 1000 s)	7	0.0136 (4 p.p.m.)
Standard	NIST SRM 2783	NIST STM 1832
Measurement time per pixel	120 s	0.1 s
Excitation	17 keV (multilayer monochromated)	17.5 keV (pink beam)
Spot size (vertical × horizontal)	500 nm × 600 nm	59 nm × 48 nm
Step size (vertical × horizontal)	1 µm × 1 µm	0.85 µm × 0.9 µm

**Table 2:** Comparison of the lower limits of detection (LLD) for Ca, Fe and Cu determined for the B16 beamline at Diamond Light Source and the P06 beamline at PETRA III as well as some of the measurement parameters For both beamlines a multi-element standard was scanned to determine the LLDs for various elements. The values for the PETRA III P06 beamline are taken from Boesenberg *et al.* (2016 ▸).

	Diamond B16	PETRA III P06
Ca *K* LLD (in ag for 1000 s)	92	2.241
Fe *K* LLD (in ag for 1000 s)	13	0.226
Cu *K* LLD (in ag for 1000 s)	7	0.109
Standard	NIST SRM 2783	AXO thin-film R11
Measurement time per pixel	120 s	20 ms
Excitation	12.7 keV	12 keV
Monochromator	Multilayer	Double-crystal monochromator
Spot size (vertical × horizontal)	500 nm × 600 nm	450 nm × 450 nm
Detector	SDD	Maia
