# Supplementary material for: Characterization of a submicro-X-ray fluorescence setup on the B16 beamline at Diamond Light Source
Source: J Synchrotron Radiat. 2018 Jun 13;25(Pt 4):1189–95. doi: 10.1107/S1600577518006203 (PMC6038595; doi:10.1107/S1600577518006203)
Supplement: Supplementary file 1 [file s-25-01189-sup1.pdf]

JOURNAL OF SYNCHROTRON RADIATION

Volume 25 (2018)

Supporting information for article:

Characterization of a submicro-X-ray fluorescence setup on the B16 beamline at Diamond Light Source

M. Rauwolf, A. Turyanskaya, D. Ingerle, N. Szoboszlai, I. Pape, A. W. Malandain, O. J. L. Fox, L. Hahn, K. J. S. Sawhney and C. Streli

Characterization of a submicro-XRF setup on the B16 beamline at the Diamond Light Source

M. Rauwolf,[a*] A. Turyanskaya,[a*] D. Ingerle,[a] N. Szoboszlai,[b] I. Pape,[c] A. W. Malandain,[c] O. J. L. Fox,[c] L. Hahn,[d] K. J. S. Sawhney[c] and C. Streli[a]

[a]*Atominstitut, TU Wien, Vienna, Austria,* [b]*Laboratory of Environmental Chemistry and Bioanalytics, Department of Analytical Chemistry, Institute of Chemistry, Eötvös Loránd University, Budapest, Hungary,* [c]*Diamond Light Source Ltd., Harwell Science & Innovation Campus, Didcot, UK, and* [d]*Karlsruhe Institute of Technology (KIT), Karlsruhe, Germany. E-mail: mirjam.rauwolf@tuwien.ac.at, anna.turyanskaya@tuwien.ac.at*

1. Supplemental data

Table 1. *Calculation of the sensitivity for 17 keV: Certified mass values for the deposition area of the filter (9.96 cm^2) were adjusted to the measured area of 36 pixels (each 500 nm × 600 nm). Net counts and fit error are given in counts per second (cps). Error propagation for devision was performed. Please be aware that due to the considerably smaller measured area (compared to the given recommendation), the sensitivity errors do not correspond to a confidence interval of 95 % and are only given as a rough indication.*

Element	Certified Values (fg)	Uncertainty (95%)	cps	2σ	Sensitivity(cps/fg)	Error Sensitivity
Ca	143.13	± 18.43	1190	± 7	8	± 1
Ti	16.16	±2.60	225	± 4	14	± 2
Cr	1.46	± 0.27	60	± 4	41	± 5
Mn	3.47	± 0.13	296	± 5	85	± 2
Fe	287.35	± 17.35	23424	± 27	82	± 5
Cu	4.38	± 0.46	669	± 4	153	± 15
Zn	19.41	± 1.41	2966	± 7	153	± 11
As	0.13	± 0.01	32	± 4	249	± 2

Table 2. *Calculation of the sensitivity for 12.7 keV: Certified mass values for the deposition area of the filter (9.96 cm^2) were adjusted to the measured area of 41 pixels (each 500 nm × 600 nm). Net counts and fit error are given in counts per second (cps). Error propagation for devision was performed. Please be aware that due to the considerably smaller measured area (compared to the given recommendation), the sensitivity errors do not correspond to a confidence interval of 95 % and are only given as a rough indication.*

Element	Certified Values (fg)	Uncertainty (95%)	cps	2σ	Sensitivity(cps/fg)	Error Sensitivity
Ca	163.0	± 21.0	3771	± 17	23	± 3
Ti	18.4	± 3.0	570	± 10	31	± 4
Cr	1.7	± 0.3	670	± 11	402	± 68
Mn	4.0	± 0.1	490	± 10	124	± 2
Fe	327.3	± 19.8	49400	± 55	151	± 9
Ni	0.8	± 0.1	130	± 7	155	± 19
Cu	5.0	± 0.5	1354	± 9	271	± 26
Zn	22.1	± 1.6	8763	± 17	396	± 28
